# P-1923. Outcomes of COVID-19 in Patients with Liver Cirrhosis

**DOI:** 10.1093/ofid/ofae631.2083

**Published:** 2025-01-29

**Authors:** Luanna Silva Monteiro Menezes, Pedro Ferrari Sales da Cunha, Magda Carvalho Pires, Lucas Rocha Valle, Rafael Lima Rodrigues Carvalho, Milena S Marcolino

**Affiliations:** Federal University of Minas Gerais (UFMG), Belo Horizonte, Minas Gerais, Brazil; Metropolitan Hospital Odilon Behrens, Belo Horizonte, Minas Gerais, Brazil; Federal University of Minas Gerais, Belo Horizonte, Minas Gerais, Brazil; Federal University of Minas Gerais (UFMG), Belo Horizonte, Minas Gerais, Brazil; Universidade Federal da Bahia, Salvador, Bahia, Brazil; Medical School, Universidade Federal de Minas Gerais, Belo Horizonte, Minas Gerais, Brazil

## Abstract

**Background:**

There is scarce data related to chronic liver disease in some developing countries, including prognosis and behavior in face of SARS-Cov-2 infection. In this context, robust studies from Latin America investigating the clinical outcomes of COVID-19 in patients with cirrhosis are lacking. Therefore, this study aimed to compare the clinical features and outcomes between COVID-infected inpatients with and without cirrhosis in Brazil.

**Methods:**

This study is a substudy of a multicenter retrospective cohort of patients from 41 Brazilian hospitals diagnosed with COVID-19 from March to September 2020 and from March 2021 to August 2022. Demographic data, clinical characteristics and outcomes were collected from the medical records. To adjust for potential confounding variables, patients with cirrhosis were matched with patients who did not have cirrhosis (controls) based on propensity score. The propensity score model was estimated by logistic regression and included sex, age, number of comorbidities and hospital of admission. Chi-square and Fisher’s exact tests were used to compare categorical variables and the Wilcoxon–Mann–Whitney test for continuous variables. Results were considered statistically significant if p-value was < 0.05.

**Results:**

This study included 749 patients (median 61 years old [interquartile range: 50-70], 63,4% were men). Among those, 117 were patients with cirrhosis and 632 matched controls. There were no significant differences in the self-reported symptoms between cases and matched controls. Compared to matched controls, cirrhotic patients presented higher in-hospital mortality (51.3% vs. 21.7%, p < 0.001), frequency of admission in intensive care unit (ICU) (51.3% vs. 38.0%, p = 0.007), invasive mechanical ventilation (IMV) (26.6% vs. 43.9%, p < 0.001), dialysis (17.9% vs. 11.1%, p = 0.038), septic shock (23.9% vs. 14.9%; p = 0.015) and institution of palliative care (19.7% vs. 7.4%; p < 0.001).

**Conclusion:**

COVID-19 inpatients with cirrhosis had significantly worse clinical outcomes when compared to matched controls. Our findings reinforce that those patients require particular attention from the healthcare team and resources.

**Disclosures:**

All Authors: No reported disclosures

